# Influence of a Nutrigenetic Intervention on Self-Efficacy, Emotions, and Rewarding Behaviors in Unhealthy Eating among Mexicans: An Exploratory Pilot Study

**DOI:** 10.3390/nu14010213

**Published:** 2022-01-04

**Authors:** Ingrid Rivera-Iñiguez, Arturo Panduro, Sergio Javier Villaseñor-Bayardo, Maricruz Sepulveda-Villegas, Claudia Ojeda-Granados, Sonia Roman

**Affiliations:** 1 Department of Molecular Biology in Medicine, Civil Hospital of Guadalajara, “Fray Antonio Alcalde”, Guadalajara 44280, Jalisco, Mexico; ingrid.riniguez@academicos.udg.mx (I.R.-I.); apanduro@prodigy.net.mx (A.P.); m_sep03@hotmail.com (M.S.-V.); claudiaojedagranados@hotmail.com (C.O.-G.); 2 Health Sciences Center, University of Guadalajara, Guadalajara 44340, Jalisco, Mexico; hijodelfraile@hotmail.com; 3 Department of Psychiatry, Civil Hospital of Guadalajara, “Fray Antonio Alcalde”, Guadalajara 44280, Jalisco, Mexico

**Keywords:** nutrigenetics, emotions, reward, self-efficacy, food behavior

## Abstract

The Genome-based Mexican (GENOMEX) diet is a strategy for preventing and managing obesity. Emotion and eating behavior in the context of a nutrigenetic intervention have not been thoroughly studied. We aimed to explore the influence of the GENOMEX diet on emotions, self-efficacy, and rewarding behaviors in unhealthy eating among subjects with risk factors for obesity-related chronic diseases. Twenty-eight subjects included in the six-month GENOMEX intervention answered questions regarding emotions that influence food consumption. Additionally, the Patient Health Questionnaire (PHQ-9) and the Reward-based eating drive scale (RED) were applied. In the study, minimal, mild, moderate, and severe depression were present in 46.4%, 39.3%, 10.7%, and 3.6%, respectively. RED did not change, but it correlated with a higher intake of fats (*r*^2^ = 0.684, β = 2.066, *p* = 0.003). Mood influenced unhealthy eating in 71.7% of subjects, and 76.9% experienced binge episodes triggered by anxiety. Sugars were the most consumed foods during binge episodes (42.2%). Both low self-efficacy levels and binge episodes were associated with high consumption of unhealthy foods. After the intervention, 10.7% of subjects reported a high level of self-efficacy. In conclusion, a culturally acceptable and genetically compatible regional Mexican food diet reduced negative emotions and unhealthy eating while increasing self-efficacy.

## 1. Introduction

Mexico presents one of the highest obesity rates in the world [1]. The national overweight and obesity prevalence in Mexico is 72.5% [2]. Conventional weight-loss interventions seem to be ineffective in the long term. Initially, people are highly motivated, but multiple factors affect their food behavior during treatment. Such factors include cultural, environmental, genetic, and psychosocial determinants [3].

In Mexico, acculturation has contributed highly to an altered food behavior. During the Spanish colonization, the native Mesoamerican staple foods were merged into the Old-World cuisine, creating traditional, regional dishes that are considered nourishing. Currently, due to the nutrition transition driven by industrialization, Mexicans have switched from eating a variety of healthy, pre-Hispanic dishes with Spanish influence to highly processed foods [4]. In addition, Mexicans socialize around food gatherings, and now, the conspicuous consumption of processed foods seems to be deeply rooted in Mexico’s food culture [5]. Furthermore, emotions, self-efficacy, and reward seem to affect food intake [6,7,8]. Self-efficacy is the confidence of an individual to perform a behavior [9]. A low self-efficacy level has been associated with lower physical activity and unhealthy food patterns [9,10]. In contrast, patients with a high level of self-efficacy have better food control and confidence in reading nutrition facts labels, eat healthy foods, and comply with dietary recommendations [11,12,13,14,15]. Food reward refers to the momentary value that is given to food by the individual at the time of consumption, and eating based on reward could potentially influence higher energy intake [16,17].

Precision nutrition interventions are innovative approaches that focus on genetically based nutrition recommendations. Our research group has suggested a Genome-based Mexican diet (the GENOMEX diet) with regional foods as a nutrigenetic strategy to diminish obesity in Mexico [18]. This diet considers the presence of adaptive gene polymorphisms related to brain reward, appetite, and energy balance in the Mexican Mestizo population of Western Mexico [19,20]. In addition, this diet is rich in fiber, antioxidants, prebiotics, and probiotic substances naturally provided in regional Mexican foods, such as maize, beans, squash, tomato, cacao, amaranth, avocado, chia seeds, and fermented beverages, among others. These foods that may contribute to a healthy microbiota have been recognized as a second brain signaling to the central nervous system, thus modulating emotions and food behavior [20]. So far, nutrigenetic dietary interventions have been implemented mainly with the “Mediterranean diet”. However, Mexican people’s genetic diversity, cultural and psychosocial contexts may interfere with eating behavior and compliance with dietary treatment [21,22].

Studies have shown the link between negative and positive emotions on food intake in adults and the influence of emotions, self-efficacy, and food behaviors while participating in weight-loss studies [23,24,25]. However, it is unknown if nutrigenetic interventions could help to improve emotional and food rewarding behaviors and self-efficacy. We hypothesized that displaying unhealthy food behavior could be ameliorated by eating a genome-based (GENOMEX) diet that considers regional Mexican foods and food culture. Therefore, the objective of this pilot study was to explore the influence of the GENOMEX diet on emotions, self-efficacy, and rewarding behaviors in unhealthy eating.

## 2. Materials and Methods

### 2.1. Study Design

The present pilot exploratory study uses data collected from the GENOMEX dietary intervention (a quasi-experimental study with a one-group pretest-posttest design). This 6-month intervention evaluated its effects (comparing baseline, three months, and six months measurements) on anthropometric and biochemical parameters and their relationship with the genetic profile in subjects with metabolic risk factors for obesity-related chronic diseases [18].

Briefly, the GENOMEX diet was designed with a macronutrient and micronutrient distribution derived from regional foods and according to the genetic background (diet-related adaptive gene polymorphisms) of the Mexican population [18,19,20]. Foods with prebiotic and probiotic activity, rich in fiber and antioxidants, such as maize, beans, squash, tomato, cacao, amaranth, avocado, chia seeds, and fermented beverages, were prescribed. This diet showed efficacy for weight loss, improvement of metabolic risk factors particularly in carriers of the methylenetetrahydrofolate reductase (*MTHFR*) 677T adaptive allele, and the acceptance of participants’ who expressed appreciation for consuming affordable foods of Mexican origin [18]. Building on these findings, we hypothesized that displaying an unhealthy food behavior could be ameliorated by eating a GENOMEX diet that considers regional foods and food culture.

In that study, dietitians explained this dietary pattern to participants, who received a weekly seasonal menu and a grocery list. In this study, no cognitive or motivational strategy was used. Patient’s compliance was assessed by follow-up phone calls between appointments. In each visit, a 24-h recall was performed, and dietary adjustments were given if necessary.

### 2.2. Participants

The GENOMEX diet study included 37 subjects aged 20–65 years from the Nutrigenetic Clinic, Department of Molecular Biology in Medicine, Civil Hospital of Guadalajara “Fray Antonio Alcalde” in Guadalajara, Jalisco, Mexico. The study was conducted from January 2016 to May 2017. Participants did not receive any type of compensation or incentive during the study. Eligibility criteria for the GENOMEX diet have been detailed previously [18]. Briefly, patients were selected if metabolic risk factors for obesity-related chronic conditions were detected without presenting overt diagnosed chronic disease. For this secondary exploratory pilot analysis, subjects were eligible if they completed the study and all questionnaires. Participants with incomplete questionnaires or who did not complete the study were excluded. Therefore, data obtained from 28 participants were considered for the present study. Written informed consent was obtained from all participants before entering the GENOMEX diet intervention. The study was performed under the ethical principles for medical research in humans (Declaration of Helsinki 2013) and approved by the Institutional Review Board of the Civil Hospital of Guadalajara “Fray Antonio Alcalde” (Ethic Approval Code 141-09).

### 2.3. Body Composition Assessment

Height was measured without shoes to the nearest 0.1 cm using a stadiometer; weight and body composition were assessed by electrical bioimpedance (In Body 3.0; Analyzer Body Composition, Biospace, Seoul, Korea). Body mass index (BMI) was the ratio of weight (kg) by height (m^2^). BMI cut-off points were as follows: normal weight (18.5–24.9 kg/m^2^) overweight (25–29.9 kg/m^2^), and obesity (>30 kg/m^2^) [26].

### 2.4. Dietary Assessment

Trained dietitians assessed participants’ dietary intake using two assessment tools at baseline, 3, and 6 months. A food frequency questionnaire (FFQ) of 64 items was applied [18]. Habitual intake was assessed with three 24-h recalls (two weekdays and one weekend day) [26] and then analyzed with Nutrikcal VO^®^ (Consinfo S.C., Mexico City, Mexico), a software containing Mexican food items. A daily average of energy and nutrients was obtained.

### 2.5. Depression Severity Assessment

The Patient Health Questionnaire (PHQ-9) was applied to assess depression symptoms. This questionnaire is a nine-item, self-reported inventory that measures symptoms occurring within the previous week [27,28]. Response options include 0 = not at all, 1 = several days, 2 = more than half days, and 3 = nearly every day. The total score can range from 0 to 27 points [28,29]. Then the severity of depression was categorized according to the cutoffs used in the validated Spanish version as follows: none or minimal = 0–4 points, mild = 5–9 points, moderate = 10–14 points, moderately severe = 15–19 points, and severe ≥ 20 [28,29].

### 2.6. Rewarding Food Behaviors

Participants also answered the Reward-Based Eating Drive Scale (RED), a 9-item self-reported scale that assesses three dimensions: lack of control, lack of satiation, and preoccupation with food. Obese subjects report those dimensions to characterize their excessive drive to eat [17]. The average score was obtained by adding the points of each answer, and the following values are assigned: 0, strongly disagree; 1, disagree; 2, neither agree nor disagree; 3, agree; and 4, strongly agree. The average score was divided by the number of sentences that make up the scale.

### 2.7. Mood and Food Behavior

We prepared a semi-structured interview to explore mood and food behavior during the study appointments. Researchers reviewed vast literature and selected key themes to create questions in a survey-like format with open or closed-ended questions [29,30]. The selected items were the frequency of unhealthy food decision making, perceived self-efficacy, which emotion triggered patients to eat more, and if patients have had binge episodes. The researchers then structured a list of close-ended questions, with open-ended follow-ups used as probes to rate each question’s frequency or intensity level. Questions were reviewed and approved by all researchers before starting the study. Table 1 shows questions and probes used to explore selected topics.

### 2.8. Data Analysis

In the GENOMEX study, a sample size of 32 was estimated using the formula for quantitative data for a single study group, with a power of 95%, an α error of 0.05, and a maximum allowed sampling error of 0.04 [36]. Thirty percent more participants were added due to possible losses during study follow-up, resulting in a sample size of 37 subjects. In the present secondary exploratory analysis, data from 28 participants who completed the questionnaires were used.

In this study, the main outcome variables were the reward score, severity of symptoms of depression, mood, and food behavior responses from close-ended questions (unhealthy food choices, self-efficacy, mood and food intake, and binge episodes). Continuous variables were expressed as mean and standard deviation, while categorical variables were expressed as frequencies and percentages. Repeated measures ANOVA, followed by Bonferroni’s post-hoc analysis was performed for data with normal distribution, such as weight, BMI, body fat%, energy, protein%, total fat%, servings of sugars, meats, refined grains, oils, milk, fruits, and vegetable servings. Data with non-normal distribution, including age, carbohydrate%, and legume servings, were analyzed with non-parametric statistics (Kruskal-Wallis and Mann-Whitney U). Frequency and percentage were calculated for the results of the questionnaires and semi-structured interviews. The χ^2^ test was used to evaluate differences between qualitative variables. Changes in the Reward-Based Eating Drive Scale were assessed with a one-way ANOVA test. A linear regression analysis was performed to assess the effect of reward on dietary intake variables as covariates. Binary logistic regression analysis was used to assess the association between unhealthy food intake (covariates) with low self-efficacy and with binge episodes (dependent variables). For this analysis, the enter method was selected, for which variables, such as dietary intake (i.e., energy, macronutrients, and food servings), were transformed into dichotomous variables, categorizing them as high intake and low intake, according to the RDA values. Reverse scores were used for the consumption of fiber and vegetables. A *p*-value of <0.05 was considered significant. Statistical analyses were performed using SPSS IBM statistical software (version 20.0; Statistical Package for the Social Sciences, Inc., Chicago, IL, USA).

## 3. Results

### 3.1. Demographic and Anthropometric Characteristics

Demographic and anthropometric measurements are shown in Table 2. In the study, there were no statistical differences in age according to BMI (*p* = 0.199).

### 3.2. Dietary Characteristics

There were no differences in dietary macronutrient distribution by BMI categories at the beginning of the study. However, obese participants consumed slightly more energy (*p* = 0.034), refined grains (*p* < 0.001), and sugar servings (*p* = 0.010), as depicted in Table 3.

Table 4 shows that, after the intervention, participants significantly decreased their energy, sugar, and milk servings consumption while increasing the number of servings of vegetables and legumes throughout the study. The number of refined grains servings decreased only in the first three months.

### 3.3. Symptoms of Depression

Table 5 shows results from the PHQ-9 questionnaire. The study detected at baseline that 53.6% of participants presented mild to severe depression symptoms. During the six-month dietary intervention, the percentage of participants with minimal symptoms of depression increased by 7.1% from baseline. Mild symptoms of depression were reduced by 3.6% at the end of the study; moderate depression slightly increased at month 3 from baseline, and symptoms of moderately severe depression were completely reduced at month 3 but not at month 6.

### 3.4. Reward-Based Eating Drive Scale

The RED scale did not change during the six-month dietary intervention (Table 6). A linear regression analysis was performed to assess the effect of reward on dietary intake variables. It was found that high scores on the RED scale correlated significantly with a “higher intake of fat” (*r*^2^ = 0.684, β = 2.066, *p* = 0.003).

### 3.5. Mood and Food Behavior

During the study, we performed a semi-structured interview with close-ended questions in order to explore mood and food behavior (Table 1) The participants’ responses were as follows:

Q1. When deciding what to eat every day, are you aware of how often you make unhealthy food choices? The percentage of “frequently” was 57.1%, which was significantly higher (*p* < 0.05) compared to the options “rarely” and “always”.

Q3. Does your mood influence your food intake? A higher proportion of participants (72.6%) affirmed that mood influences their food intake (*p* < 0.05).

Q4. In what way does your mood influences your food intake? The percentage of participants that responded “eat” more” (71.7%) was significantly higher (*p* < 0.05) compared to the response options “eat less” and “do not eat”.

Q5. Have you experienced a binge episode? A total of 76.9% of participants confirmed that they experience binge episodes.

Q6. What triggers the binge episode? A total of 40% of participants responded that binge episodes were triggered by anxiety, 13% by social eating, 20% by emotions, and 26.7% by other circumstances.

Q7. What flavor induces you to binge eat? During these episodes, participants responded that the most selected flavors were sugary at 42.2% (*p* < 0.05), followed by fatty at 28.9%, and salty foods at 28.9%.

### 3.6. Changes in Food Decisions and Self-Efficacy during the Dietary Intervention

We found no significant differences in the percentage of change in the frequency of unhealthy food decisions. However, we found that the percentage of participants that answered “rarely” about making unhealthy food decisions increased by 21.4% at the end of the study. In comparison, the number of participants who responded “frequently” was reduced by 7.1%, and those who responded “always” were reduced entirely.

The self-reported low self-efficacy was reduced by 10.7% during the study; the moderate self-efficacy level increased 7% at month-3 but did not change at month-6 from baseline. The high self-efficacy level increased by 11.3% at the end of the study. However, these changes were not statistically significant.

### 3.7. Risk of Unhealthy Food Consumption with a Low Level of Self-Efficacy and the Presence of Binge Episodes

The associations between unhealthy food intake with low self-efficacy and binge episodes was evaluated by a binary logistic regression analysis (Table 7). A high-frequency consumption of fats, saturated fats, and low consumption of vegetables and fiber were associated with low self-efficacy. A high consumption of servings of fats, refined grains, meats, grams of sugar, high energy intake, and a high percentage of carbohydrates, fats, saturated fats, and proteins were associated with binge-eating episodes.

## 4. Discussion

In Mexico, overweight and obesity are leading public health problems. Mexicans with these health conditions constantly struggle to maintain long-term dietary changes. This study explored how emotions, self-efficacy, and rewarding behaviors interfere with following a healthy diet during a nutrigenetic intervention. We found that negative emotional factors, such as anxiety, food consumption for reward, and a low level of self-efficacy, predispose Mexicans to consume foods high in fat and sugar. In addition, these factors triggered binge-eating behaviors. However, during a GENOMEX dietary intervention, improvements in unhealthy eating decisions, and higher self-efficacy were found.

In the GENOMEX diet, we recruited participants with metabolic abnormalities no matter the BMI value since it has been reported that normal-weight patients also consume an inadequate hepatopathogenic dietary pattern [37,38]. This diet refers to higher consumption of more industrialized, calorie-dense foods and high amounts of saturated fatty acids, cholesterol, and high-fructose foods as well as lower consumption of polyunsaturated fatty acids (ω-3/ω-6), fiber, and micronutrients with antioxidant properties, which leads to metabolic abnormalities [37]. However, the small proportion of normal-weight patients included in the study may be due to Mexico’s general excess weight problem. Interestingly, all participants regardless of BMI consumed excessive energy, but people with obesity tended to consume more calories and refined grains than the rest of the participants. Normal-weight patients consumed fewer sugar servings. This finding is similar to what has been reported in previous studies, in which unhealthy food patterns were consumed highly among people with higher BMI [39]. However, during the study, participants decreased their energy intake, sugar, dairy servings, and refined grains and increased the number of servings of vegetables and legumes.

Likewise, emotional factors could be driving unhealthy dietary patterns among West Mexicans. The PHQ-9 questionnaire revealed that 53.6% of participants had mild to severe depression symptoms at the beginning of the study, which gradually decreased. Therefore, a higher proportion of participants with minimal depressive symptoms was detected at the end of the intervention. Similar studies have also reported lower depression symptoms during diet interventions [40,41]. Moreover, we assessed if participants were eating based on rewards. We performed a linear regression analysis to assess the effect of reward on dietary intake and we found that a high RED score correlated with a higher fat intake. Other researchers have found that the RED score correlates with BMI and cravings for energy-dense foods coded as savory and sweet foods [17,42]. In the present study, there was a higher average RED score of 1.26 (SD = 0.81) compared to those previously reported, in which the average RED score was 0.40 to 0.77 and ranged from 0 to 1.89 [17,42]. However, it is plausible that the small sample size could have limited our results.

Additionally, to explore emotional behaviors that contribute to the unhealthy eating of participants during the GENOMEX dietary intervention, we conducted close-ended questions with open-ended follow-ups. As a result, 57.1% of participants considered making frequently unhealthy decisions about their food. Similar findings by other researchers show that patients with obesity made more impulsive unhealthy food decisions in the presence of food and hunger [43,44]. Regarding mood and food behavior, 72.6% of participants affirmed that mood influences their food intake, and 71.7% of participants stated that mood prompted them to eat more. Van Strien and colleagues proposed that higher intake is experienced after feelings of sadness [33]. However, it is not clear if feelings of happiness or even neutral moods could influence a higher intake of foods [25].

Interestingly, we were able to identify that 76.9% of participants confirmed to experience binge episodes triggered by anxiety in 40% of the occasions. Associations between anxiety and binge eating have been demonstrated [6,7]. Furthermore, research from ecological momentary assessment (EMA) studies, which evaluate variables of interest in the natural environment in real time, indicate that increasing negative emotions trigger binge-eating episodes [45,46]. This study found that participants reported consuming sugary foods during these episodes at 42.2%, followed by fried foods at 28.9%, and salty foods at 28.9%. We also found that high consumption of servings of fats, refined grains, red meats, grams of sugar, and high energy intake and a high percentage of carbohydrates fats, saturated fats, and proteins were associated with a high risk of binge episodes. This can be explained by the combination of sugary and fatty foods that may generate positive feedback in the brain reward system, triggering overeating [47].

A high level of self-efficacy has been associated with higher adherence to dietary treatment and healthy eating habits [9,10,11,12]. In the present study, we found increases in self-efficacy and that a low self-efficacy level was significantly associated with high-frequency consumption of total fats, saturated fats, and a high risk of low consumption of vegetables and fiber (Table 7). Similarly, changes in self-efficacy level associated with decreases in dietary fat intake in low-income subjects with excess weight have been reported [48]. Furthermore, a positive correlation between fruit and vegetable intake and high self-efficacy among African American women was reported [49]. A meta-analysis that assessed behavior techniques to promote self-efficacy found that dietary interventions focusing on self-monitoring, feedback on performance, revision of behavioral goals, provided rewards, or planned social support increased dietary self-efficacy significantly more than interventions that did not [50]. Moreover, in a pilot study, families that received a weekly free meal kit during 10 weeks with educational preparation tips found increases in self-efficacy [51]. Additionally, in a Mediterranean diet study, a positive relationship between dietary adherence and self-efficacy were found [52]. Therefore, we considered clinically important the increments in high self-efficacy level found in the present study because it could help patients to adhere to dietary guidelines and to meet their needs.

In the GENOMEX study, we were focused on the metabolic changes related to the nutrient-gene interactions of the prescribed foods. We did not provide psychological or adherence reinforcement techniques to promote self-efficacy, like the ones used in the social cognitive theory or the transtheoretical model. These strategies have been previously reported with positive results [50,53]. Such strategies include motivation, reinforcements, and working on participants’ skills and are based on the social cognitive theory, which focuses on the influence of individual experiences, the actions of others, and environmental factors on individual health behaviors [54]. In this exploratory pilot study, we did not test the social cognitive theory by describing the participant´s experiences, expectations, or if they developed skills during the intervention that could influence the present results. In addition, we did not evaluate the stages of change of the participants, which is proposed by the transtheoretical model. The transtheoretical model is a guideline to promote positive health behavior changes; it aims to understand individual’s behavioral changes and to describe the process of those changes. The constructs in this model are stages of change (precontemplation, contemplation, preparation, action, maintenance, and termination), self-efficacy, decisional balance, and processes of change. [55]. It is suggested that as subjects advance through those stages, it is more likely that they perceive more benefits than disadvantages from adopting positive behavior changes when compared to the initial stages.

Nonetheless, during the GENOMEX intervention, we were able to find that symptoms of depression decreased along with unhealthy food decisions. It is plausible that the characteristics of the diet plus the different menus and grocery lists used in the study might have contributed to these positive results. We prescribed foods according to the participants’ genetic and cultural background (beans, corn tortilla, amaranth, chia and pumpkin seeds, tomato, nopal, quelites (regional leafy greens), and avocado) [18]. A plausible explanation of the changes in mood and self-efficacy could be because this diet also considers the genetic polymorphisms involved to appetite control and energy balance among the Mexican population. Likewise, the prescribed regional foods were rich in fiber and antioxidants with prebiotic and probiotic activity. These foods have been well described to ameliorate gut microbiota performance in terms of mood and cognition by maintaining a bidirectional communication with the central nervous system [20]. Therefore, it could be possible that the consumption of these prebiotics/probiotics ameliorated unhealthy food behavior and negative emotions by restoring the gut-brain axis. However, these results cannot be generalized because nutrition precision strategies need to be individualized in the context of cultural and regional background of a specific population.

Finally, some limitations in this study were a small sample size since we only considered data from participants who completed the questionnaires during the GENOMEX intervention. Likewise, we were not able to recruit a control group to compare these results with healthy subjects. Additionally, we recognize that the unfeasibility of using a validated questionnaire related to emotions, self-efficacy, and food behavior at each of the subjects’ appointment could limit the present results. However, the strengths of this study were that we selected the main topics that we were interested in to explore the frequency of unhealthy food decisions, perceived self-efficacy, and which emotions triggered patients to eat more and if patients had binge episodes. Furthermore, we used the Patient Health Questionnaire and Reward-Based scale to question the participants about their emotions and self-efficacy level during the study. Additionally, we detected symptoms of depression, low level of self-efficacy, and high rewarding inputs that elicit unhealthy food behaviors. Psychologists usually do not refer these patients to dietitians or recommend making changes in their diet; however, this study provided them with the opportunity to restore their emotions by consuming a genome-based diet. The results presented here will allow us to design further nutrigenetic studies focused on the relationship between polymorphisms of food intake, satiety, and reward genes and food behavior traits.

## 5. Conclusions

In this study, negative emotions influenced unhealthy food decision making and low self-efficacy. The combination of sugary/fatty/salty foods consumed by Mexicans during negative emotional experiences disrupts the traditional food culture. However, consuming foods compatible with their genetic background seems to decrease unhealthy eating decisions and increase self-efficacy. This study supports the use of personalized nutrition that uses the genetic information as a strategy to enhance adherence and highlights the importance of designing lifestyle interventions that address the process of food decision-making, self-efficacy, mood, and emotions. Further research is needed to associate genetic factors, such as gene polymorphisms, with food behavior traits.

## Figures and Tables

**Table 1:** Close-Ended Questions used in the GENOMEX dietary intervention.

Topics	Questions (Q)	Probes	References
Frequency of unhealthy food decision making	Q1. When deciding what to eat every day, are you aware of how often you make unhealthy food choices? Rarely, Frequently, or Always?	Describe examples of those times (food shopping, food preparation, snaking, travels)	[31]
Perceived self-efficacy	Q2. How confident do you feel about following a healthy diet? Low, Moderate, or High?	Explain that self-efficacy means how confident is a person to perform a behavior (following a healthy diet)	[13,14,15,32]
Mood and food intake	Q3. Does your mood influence your food intake?	Discuss if the participant considers being an emotional eater	[8,33]
	Q4. In what way your mood influences your food intake?	Discuss if mood makes participant eat more, eat less, or not eat	
Binge episodes	Q5. Have you experienced a binge episode?	Explain that the episode characterizes by eating large quantities of food rapidly to the point of discomfort and without control	[6,7,34,35]
	Q6. What triggers the binge episode? Q7. What flavor induces you to binge eat?	Describe and discuss with participant instances that could trigger binge episodes and ask for the most frequent	

**Table 2:** Demographic and anthropometric characteristics of subjects (*n* = 28) enrolled in the GENOMEX intervention.

Variable	Normal Weight	Overweight	Obesity
Subjects *n* (%)	5 (17.85)	11 (39.29)	12 (42.86)
Age (years)	51 ± 4.24	43.64 ± 11.87	39.58 ± 13.07
Weight (kg)	60.12 ± 6.83	70.38 ± 5.81	90.93 ± 16.6
BMI (kg/m^2^)	23.72 ± 0.48	26.90 ± 1.20	34.37 ± 4.15
Body fat (%)	31.02 ± 1.82	31.15 ± 5.23	40.08 ± 4.51

Data are expressed as mean ± standard deviation unless indicated otherwise. GENOMEX, genome-based Mexican diet; BMI, body mass index.

**Table 3:** Baseline dietary characteristics of subjects (*n* = 28) enrolled in the GENOMEX intervention according to BMI.

Variable	Normal Weight (*n* = 5)	Overweight (*n* = 11)	Obese (*n* = 12)	*p*
Energy (kcal/day)	2047.80 ± 637.16	2053.20 ± 720.10	2510.97 ± 770.93	0.034 ^a^
Proteins (%)	21.60 ± 7.50	16.90 ± 4.94	17.53 ± 4.55	0.152 ^a^
Total fat (%)	32.20 ± 4.91	33.10 ± 8.36	31.41 ± 8.15	0.755 ^b^
Carbohydrates (%)	49.40 ± 10.64	52.45 ± 10.52	52.71 ± 7.81	0.744 ^b^
Sugars servings	1.33 ± 1.22	4.95 ± 4.26	6.66 ± 4.80	0.010 ^a,^*
Meats servings	8.53 ± 6.34	7.17 ± 5.75	9.28 ± 4.96	0.377 ^a^
Refined grains servings	7.71 ± 2.47	7.42 ± 2.41	11.37 ± 3.99	<0.001 ^a,^**
Oils servings	2.10 ± 1.59	3.83 ± 2.80	5.18 ± 4.09	0.132 ^a^
Milk servings	1.14 ± 0.86	0.93 ± 0.81	0.75 ± 1.17	0.663 ^a^
Fruits servings	3.80 ± 2.96	2.94 ± 2.06	2.41 ± 1.96	0.321 ^a^
Vegetables servings	3.43 ± 3.51	2.97 ± 2.05	3.34 ± 2.56	0.850 ^a^
Legumes servings	0.56 ± 0.95	0.30 ± 0.41	0.77 ± 1.04	0.258 ^b^

Data are expressed as Mean ± standard deviation. GENOMEX, genome-based Mexican diet; Kcal/day, kilocalories per day. * Normal weight vs. Overweight and Obese. ** Obese vs. Overweight. ^a^ Statistical analysis was conducted with one-way ANOVA, followed by Bonferroni’s post-hoc. ^b^ Kruskal-Wallis test and Mann-Whitney U.

**Table 4:** Dietary changes of subjects enrolled in the GENOMEX intervention.

Variable	Baseline (*n* = 28)	3 Months (*n* = 28)	6 Months (*n* = 28)	*p*
Energy (kcal/day)	2238.74 ± 712.97	1498.13 ± 418.59 *	1748.58 ± 639.93 **	0.002
Proteins (%)	17.74 ± 4.06	19.96 ± 5.17	23.90 ± 27.41	0.182
Total fat (%)	31.06 ± 7.95	28.21 ± 9.16	36.70 ± 26.50	0.192
Carbohydrates (%)	53.64 ± 8.98	54.63 ± 10.57	50.93 ± 9.31	0.160 ^a^
Sugars servings	5.12 ± 4.38	1.52 ± 2.02 *	1.63 ± 1.90 **	<0.001
Meats servings	7.78 ± 4.40	5.74 ± 3.20	7.00 ± 2.77	0.377
Refined grains servings	9.28 ± 3.85	6.57 ± 2.74 *	7.48 ± 8.27	0.003
Oils servings	4.31 ± 3.88	2.62 ± 1.95	3.45 ± 4.37	0.437
Milk servings	1.03 ± 1.18	0.30 ± 0.60 *	0.18 ± 0.39 **	<0.001
Fruits servings	3.16 ± 2.02	2.60 ± 1.94	3.43 ± 2.06	0.527
Vegetables servings	3.04 ± 2.61	5.05 ± 3.37 *	5.95 ± 3.56 **	<0.001
Legumes servings	0.63 ± 0.98	0.68 ± 1.17 *	0.75 ± 0.93 **	<0.001 ^a^

Data are expressed as mean ± standard deviation. GENOMEX, Genome-based Mexican diet. deviation; Kcal/day, kilocalories per day. * Basal vs. 3 Months. ** Basal vs. 6 Months. Analysis conducted with repeated measures ANOVA, followed by Bonferroni’s post-hoc. ^a^ Kruskal–Wallis test.

**Table 5:** Severity of depression symptoms of subjects (*n* = 28) enrolled in the GENOMEX intervention.

Severity of Depression Symptoms	Baseline *n* (%)	3 Months *n* (%)	6 Months *n* (%)	Change (%)	*p* ^a^
Minimal	13 (46.4)	12 (42.9)	15 (53.5)	15.3	0.285
Mild	11 (39.3)	11 (39.3)	10 (35.7)	−9.2	0.402
Moderate	3 (10.7)	5 (17.8)	2 (7.14)	−33.3	0.352
Moderately severe	1 (3.6)	0	1 (3.5)	−2.7	0.495

GENOMEX, genome-based Mexican diet. ^a^ Student’s *t*-test was used for comparisons: Baseline vs. 6 Months.

**Table 6:** Reward scale score of subjects (*n* = 28) enrolled in the GENOMEX intervention.

Reward Scale	Baseline (*n* = 28)	3 Months (*n* = 28)	6 Months (*n* = 28)	*p*
Score	1.26 ± 0.81	0.94 ± 0.62	1.05 ± 0.069	0.368 ^a^

Data are expressed as mean ± standard deviation. GENOMEX, genome-based Mexican diet. *n*. ^a^ One-way ANOVA.

**Table 7:** Association of unhealthy eating with low self-efficacy level and binge-eating episodes among subjects enrolled in the GENOMEX dietary intervention.

**Low Self-Efficacy Level ^a^**		
**Characteristic**	**OR, 95% CI**	***p***
High consumption of fats	10.89, 1.91–62.15	0.005
High consumption of saturated fats	1.257, 1.001–1.577	0.049
Low consumption of vegetables	1.671, 1.191–2.345	0.003
Low consumption of fiber	1.093, 1.015–1.178	0.019
**Binge-Eating Episodes ^b^**		
**Characteristic**	**OR, 95% CI**	***p***
High consumption of fats (servings)	1.221, 1.053–1.416	0.008
High consumption of refined grains (servings)	1.134, 1.051–1.223	0.001
High consumption of meats (servings)	1.160, 1.058–1.272	0.002
High consumption of sugars (servings)	1.192, 1.053–1.349	0.006
High consumption of sugars (gr)	1.023, 1.009–1.038	0.002
High energy consumption (kcal)	1.000, 1.000–1.001	0.002
High consumption of carbohydrates (%)	1.022, 1.008–1.035	0.001
High consumption of fats (%)	1.038, 1.015–1.062	0.001
High consumption of saturated fats (%)	1.135, 1.049–1.228	0.002
High consumption of proteins (%)	1.066, 1.024–1.109	0.002

GENOMEX, genome-based Mexican diet; OR, odds ratio; CI, confidence interval. Hosmer-Lemeshow test: ^a^ chi-square = 4.7, *p* = 0.03; Nagelkerke R square = 0.155. ^b^ chi-square = 3.9, *p* = 0.025; Nagelkerke R square = 0.175.

## Data Availability

The data presented in this study are available on request from the corresponding author. The data are not publicly available due to policies of patients ‘data privacy.
